# Anti-thymocyte globulin-resistant CD4^+^ memory T cells contribute to haplo-fever after allogeneic hematopoietic stem cell transplantation

**DOI:** 10.1097/BS9.0000000000000280

**Published:** 2026-02-26

**Authors:** Huihong Huang, Ting Lu, Honglin Duan, Jingtao Huang, Quan Gu, Hui Cheng, Zengkai Pan, Xiaoxia Hu

**Affiliations:** a Shanghai Institute of Hematology, State Key Laboratory of Medical Genomics, National Research Center for Translational Medicine at Shanghai, Ruijin Hospital, Shanghai JiaoTong University School of Medicine, Shanghai, China; b State Key Laboratory of Experimental Hematology, National Clinical Research Center for Blood Diseases, Haihe Laboratory of Cell Ecosystem, Institute of Hematology & Blood Diseases Hospital, Chinese Academy of Medical Sciences & Peking Union Medical College, Tianjin, China; c Collaborative Innovation Center of Hematology, Shanghai JiaoTong University School of Medicine, Shanghai, China

**Keywords:** Graft-versus-host disease, Haplo-fever, Hematopoietic stem cell transplantation, Measurable residual disease, Memory T cell

## Abstract

Haplo-fever (HF) is a form of non-infectious fever that occurs shortly after haploidentical (HID) hematopoietic stem cell transplantation (HSCT). Its clinical significance, risk factors, and pathophysiology, particularly in the context of anti-thymocyte globulin (ATG)-based prophylaxis for graft-versus-host disease (GvHD), remain largely unknown. Here, we retrospectively analyzed clinical data from 143 consecutive HID HSCT recipients. Patients with HF exhibited a higher incidence of chronic GvHD, a lower risk of measurable residual disease recurrence, and improved event-free survival, particularly among those receiving ATG-based acute GvHD prophylaxis. Identified risk factors for HF included higher infused doses of CD34^+^ cells, mononucleated cells, CD3^+^ T cells, and CD4^+^ memory T cells. At 90 days post-transplant, patients with HF demonstrated elevated absolute numbers of CD4^+^ memory T cells and activated CD4^+^ and CD8^+^ T cells. RNA sequencing of peripheral blood CD3^+^ T cells at day 2 post-transplant revealed transcriptional signatures of T-cell activation and an abundance of activated CD4^+^ memory T cells as hallmarks of HF. Using multiple complementary approaches, we demonstrated the impact of HF on transplant outcomes, identified its risk factors, and elucidated the underlying cellular and molecular mechanisms.

## 1. INTRODUCTION

Allogeneic hematopoietic stem cell transplantation (allo-HSCT) is a potentially curative treatment for hematologic malignancies. With the introduction of post-transplantation cyclophosphamide (PTCy) and anti-thymocyte globulin (ATG) for graft-versus-host disease (GvHD) prophylaxis, haploidentical (HID) HSCT has achieved outcomes comparable to those of matched donor HSCT.^1–3^ Non-infectious fever occurring within the first 5 days following graft infusion^4,5^ (termed haplo-fever, HF) has emerged as an important clinical issue in HID HSCT. Indeed, HF is significantly more frequent among patients receiving PTCy-based prophylaxis (~90%)^6^ compared to those receiving ATG (~38%).^7^ With the widespread adoption of HID HSCT, understanding the incidence, risk factors, and clinical significance of HF has become increasingly important.

Several retrospective studies have identified potential HF risk factors in PTCy-based HID HSCT, including older age, prior radiation therapy, human lymphocyte antigen (HLA) class II mismatch, granulocyte colony-stimulating factor (G-CSF)–mobilized peripheral blood stem cell (PBSC) grafts, and conditioning intensity.^4,8–10^ Nevertheless, the clinical impact of HF on transplant outcomes remains controversial. Some reports have associated HF with increased risk of engraftment syndrome,^7^ grade II–IV acute GvHD,^7^ non-relapse mortality (NRM),^10^ and inferior 1-year overall survival (OS), disease-free survival (DFS),^11^ and relapse-free survival (RFS).^12^ By contrast, Solh et al^5^ found no significant effect of HF on survival or GvHD. Moreover, in adult patients undergoing HID HSCT with ATG-based GvHD prophylaxis, the risk factors and consequences of HF are still poorly defined.

In addition to the limited understanding of its risk factors and clinical impact, the pathophysiology of HF is also poorly understood. HF is considered a cytokine-mediated syndrome, resembling the cytokine release syndrome observed in patients receiving chimeric antigen receptor (CAR) T cell therapy, with serum interleukin (IL)-6 levels typically peaking around day 2 and normalizing by day 5.^13^ The higher incidence of HF in PBSC transplants may result from the greater number of T lymphocytes in peripheral blood grafts compared to bone marrow (BM).^14^ It has been hypothesized that the activation and proliferation of alloreactive T cells upon antigen exposure,^8,11^ along with activation of proinflammatory cells such as monocytes and dendritic cells,^15,16^ contribute to HF; however, the specific T-cell subsets and their functional status are poorly characterized.

In this study, we investigated the incidence and risk factors of HF in recipients of HID HSCT with ATG-based acute GvHD prophylaxis and evaluated its impact on transplant outcomes. Given the critical role of T cells in the development of cytokine release syndromes, we also examined the transcriptomic profile of T cells on day 2 post-transplantation to elucidate the molecular mechanisms underlying HF.

## 2. MATERIALS AND METHODS

### 2.1. Patients and study design

This was a single-center, retrospective study based on the transplant database of Shanghai Ruijin Hospital (chictr.org.cn identifier: ChiCTR2200063705). Consecutive patients who underwent allo-HSCT between April 2021 and April 2023 were screened. The inclusion criteria were as follows: age ≥16 years; recipient of HID HSCT; and an expected survival time ≥30 days. Exclusion criteria included: age <16 years and incomplete medical records. The final follow-up was conducted on July 30, 2025. The study was approved by the institutional review board of Ruijin Hospital (approval number: 2021-388) and conducted in accordance with the *Declaration of Helsinki*.

### 2.2. Transplant regimen and GvHD prophylaxis

Preconditioning regimens were administered as previously described.^17,18^ A total of 134 patients (93.7%) received a myeloablative conditioning regimen, while 9 patients (6.3%) received a reduced-intensity regimen. All patients received granulocyte colony-stimulating factor (G-CSF)–mobilized PBSCs from HID donors. GvHD prophylaxis included either 10 mg/kg ATG (Sanofi, France)^19,20^ or 100 mg/kg PTCy,^21^ following previously established protocols. ATG was administered to 71 patients, and PTCy to 72 patients.

### 2.3. MRD monitoring protocol

Measurable residual disease (MRD) was monitored at 1, 2, 3, 4.5, 5, 6, 9, and 12 months post–allo-HSCT, and every 6 months thereafter.^22–24^ MRD was assessed using multiparameter flow cytometry (MFC) based on leukemia-associated aberrant immunophenotypes (LAIPs) and/or “different-from-normal” (DfN) patterns. For acute myeloid leukemia (AML), MRD positivity was defined as levels >0.1%, whereas for acute lymphoblastic leukemia (ALL), the threshold was >0.01.^25,26^

### 2.4. Definitions of transplant events

HF was defined as a temperature ≥38°C occurring between day 0 and day +5 post-transplant, in the absence of identifiable causative pathogens, and considered related to graft infusion.^5^ At the onset of fever, all patients received empiric broad-spectrum antibiotics.

Neutrophil engraftment was defined as the first of 3 consecutive days with an absolute neutrophil count >0.5 × 10^9^/L. Platelet engraftment was defined as a platelet count >20,000/μL for 7 consecutive days without transfusion support. Relapse was defined as the recurrence of BM blasts >5%, the reappearance of blasts in peripheral blood, development of extramedullary disease, or recurrence of pre-transplantation chromosomal abnormalities. Epstein–Barr virus (EBV) or cytomegalovirus (CMV) reactivation was defined as >1 × 10^3^ IU/mL of viral DNA in plasma. NRM was defined as death in the absence of disease progression or relapse. OS was defined as time from transplant to death from any cause. For EFS, events included MRD recurrence, relapse, or death. GvHD-relapse-free survival (GRFS) was calculated from the day of transplant to the occurrence of grade III–IV acute GvHD (aGvHD), moderate to severe chronic GvHD (cGvHD), relapse, or death from any cause. Leukemia-free survival (LFS) was defined as survival without evidence of relapse at any time post-transplant. Patients without events were censored at the time of last follow-up.

### 2.5. Flow cytometric analysis

Peripheral blood nucleated cells were obtained by lysing red blood cells. Multicolor flow cytometric analysis was conducted using the BD FACSymphony A5 to characterize immune cell subsets. Antibodies were obtained from BD Pharmingen, including: Fixable Viability Stain 440UV (Cat# 566332), anti-CD45 BUV395 (Cat# 563792), anti-CD45RO PE-Cy7 (Cat# 337168), anti-CD3 BUV496 (Cat# 612940), anti-CD4 APC-Cy7 (Cat# 341095), anti-CD8 BV510 (Cat# 563919), anti-CD19 APC (Cat# 555415), anti-CD56 FITC (Cat# 340410), and anti-CD28 PerCP-Cy5.5 (Cat# 337181).

### 2.6. RNA-Seq of CD3^+^ T cells

CD3^+^ T cells were isolated from peripheral blood nucleated cells of 15 HSCT cases (9 patients with HF and 6 patients without HF) via magnetic bead sorting using the EasySep Human T Cell Isolation Kit (StemCell Technologies, Cat# 17951). Cell viability and concentration were assessed using the Countstar platform (Aber Instruments Ltd). Total RNA from CD3^+^ T cells was extracted for RNA sequencing. Libraries were generated using the Smart-seq method and sequenced on the Illumina NovaSeq platform. Reads were aligned to the human genome (hg38) using the STAR package, and unique, properly paired reads were filtered using Samtools. Gene expression count tables were generated using HTSeq. Differential expression analysis and normalization were performed using DESeq2, with fold change (FC) ≥2 and adjusted *p* < 0.050 considered statistically significant. Gene Ontology (GO) enrichment was analyzed using Metascape. Differential expression results were visualized with pheatmap and EnhancedVolcano. Gene set enrichment analysis (GSEA) was used to identify significant gene sets. Immune cell profiles were estimated from RNA-seq data using CIBERSORTx (https://cibersortx.stanford.edu/).

### 2.7. Statistical analysis

Continuous variables were summarized as medians with ranges (or standard deviations, SD, where applicable). Associations between patient characteristics and fever status were assessed using the Fisher exact test for categorical variables and the Wilcoxon rank-sum test for continuous variables. The Kaplan–Meier method was used to estimate OS, EFS, and GRFS with the log-rank test. and the cumulative incidences of EBV, CMV, aGvHD, cGvHD, and MRD recurrence were analyzed using Gray test with non-relapse death as the competing event.

Univariate and multivariable logistic regression and Cox proportional hazards models were applied to evaluate risk factors for HF and HF-related transplant outcomes, respectively, including all those previously reported to be potentially associated with these endpoints. For outcomes with competing risks, such as GvHD or MRD recurrence, Fine-Gray subdistribution hazards models were applied. Causal mediation analysis of HF (independent variable) and cGvHD (mediator) was conducted using the quasi-Bayesian method. *p* Values <0.050 were considered statistically significant. Analyses were performed using GraphPad Prism (version 9.0) and the R statistical software package (www.rproject.org).

## 3. RESULTS

### 3.1. Patient characteristics

**Figure 1.**
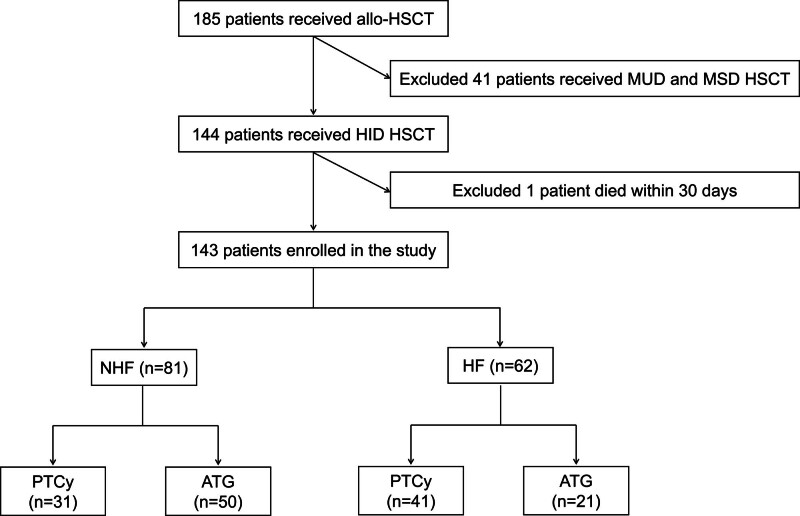
Flowchart of the study design. ATG = anti-thymocyte globulin, HF = haplo-fever, HID = haploidentical, HSCT = hematopoietic stem cell transplantation, MSD = matched sibling donor, MUD = matched unrelated donor, NHF = non-HF, PTCy = post-transplantation cyclophosphamide.

A total of 185 patients underwent allo-HSCT at our center between April 2021 and April 2023. Of these, 144 underwent HID HSCT; after excluding 1 patient who died within 30 days post-transplant, 143 consecutive patients were included in this study (**Fig. 1**, **Table 1**).

**Table 1:** Baseline characteristics of patients in NHF and HF groups in the entire cohort.

Characteristics	Entire cohort (n = 143)	NHF group (n = 81)	HF group (n = 62)	*p* Value
Median age, y (range)	48 (15–69)	48 (16–67)	48 (15–69)	0.880
Sex, n (%)				0.649
Male	73 (51.0)	40 (49.4)	33 (53.2)	
Female	70 (49.0)	41 (50.6)	29 (46.8)	
Follow-up duration in days, median (range)	1001 (137–1522)	880 (137–1522)	1160 (161–1513)	<0.001
Underlying disease, n (%)				0.583
Myeloid disease	98 (68.5)	54 (66.7)	44 (71.0)	
Lymphoid disease	45 (31.5)	27 (32.3)	18 (29.0)	
HCT-CI scores, n (%)				0.343
0 (low risk)	106 (74.1)	58 (71.6)	48 (77.4)	
1–2 (intermediate risk)	28 (19.6)	19 (23.5)	9 (14.5)	
≥3 (high risk)	9 (6.3)	4 (4.9)	5 (8.1)	
Conditioning, n (%)				
MAC	134 (93.7)	77 (95.1)	57 (91.9)	0.446
RIC	9 (6.3)	4 (4.9)	5 (8.1)	
Blood group disparity, n (%)				0.282
Matched	54 (50.9)	30 (57.7)	24 (44.4)	
Major mismatched	25 (23.6)	9 (17.3)	16 (29.6)	
Minor mismatched	18 (17.0)	10 (19.2)	8 (14.8)	
Major and minor mismatched	9 (8.5)	3 (5.8)	6 (11.1)	
DSA, n (%)				0.668
Negative	88 (83.0)	44 (85.6)	44 (81.5)	
Positive	18 (17.0)	8 (15.4)	10 (18.5)	
GvHD prophylaxis, n (%)				0.001
ATG	71 (49.7)	50 (61.7)	21 (33.9)	
PTCy	72 (50.3)	31 (38.3)	41 (66.1)	
Disease status, n (%)				0.648
Remission	122 (85.3)	70 (86.4)	52 (83.8)	
Active	21 (14.7)	11 (13.6)	10 (16.1)	
MNC counts in graft, median (range, ×10^8^/kg)	13.5 (4.9–25.6)	12.8 (4.9–23.4)	14.9 (6.3–25.6)	0.026
CD34^+^ cell counts in graft, median (range, ×10^6^/kg)	9.2 (5.3–15.4)	8.8 (5.3–14.5)	9.8 (6.0–15.4)	0.008

ATG = anti-thymocyte globulin, DSA = donor-specific antibody, GvHD = graft-versus-host disease, HCT-CI = Hematopoietic Cell Transplantation Comorbidity Index, HF = haplo-fever, MAC = myeloablative conditioning, MNC = mononuclear cell, NHF = non-haplo-fever, PTCy = post-transplantation cyclophosphamide, RIC = reduced-intensity conditioning.

The median follow-up was 1001 days (range: 137–1522 days). During the follow-up period, 18 patients experienced relapse, and 10 patients died from NRM. For the entire cohort, the 3-year probability of relapse, NRM, LFS, and OS were 13.4% (95% confidence interval [CI]: 7.4%–19.0%), 7.3% (95% CI: 2.8%–11.5%), 76.2% (95% CI: 69.3%–83.7%), and 87.3% (95% CI: 81.9%–93.2%), respectively. The cumulative incidences of aGvHD, cGvHD, CMV, and EBV reactivation are detailed in Table 2.

**Table 2:** Transplant outcomes.

Transplant outcomes	Entire cohort (n = 143)	NHF group (n = 81)	HF group (n = 62)	*p* Value
CIs of aGvHD, incidence (95% CI)				
I–IV	29.5 (22.7–37.7)	23.5 (15.7–34.4)	37.2 (26.5–50.4)	0.054
II–IV	20.6 (15.0–28.1)	13.6 (7.8–23.2)	27.4 (18.0–40.3)	0.037
III–IV	7.0 (3.8–12.6)	6.2 (2.6–14.2)	8.1 (3.5–18.2)	0.651
CIs of cGvHD, incidence (95% CI)				
Total cGvHD	40.9 (37.3–59.9)	35.0 (17.1–48.5)	63.1 (45.9–74.9)	0.008
m/s cGvHD	21.3 (12.7–29.0)	18.7 (4.3–30.9)	25.8 (13.6–36.2)	0.142
CIs of CMV reactivation, incidence (95% CI)	58.1 (50.2–66.4)	54.0 (43.4–65.3)	61.3 (49.5–73.3)	0.374
CIs of EBV reactivation, incidence (95% CI)	45.4 (37.2–54.5)	48.6 (38.0–60.4)	40.5 (42.4–53.9)	0.271
CIs of relapse, incidence (95% CI)	13.4 (7.4–19.0)	17.0 (8.1–25.0)	8.7 (1.1–15.8)	0.140
CIs of MRD recurrence, incidence (95% CI)	19.8 (12.6–26.4)	26.2 (15.0–35.9)	12.0 (3.2–20.0)	0.053
NRM, incidence (95% CI)	7.3 (2.8–11.5)	9.1 (2.4–15.4)	4.9 (0–10.2)	0.371
OS, probability (95% CI)	87.3 (81.9–93.2)	84.3 (76.5–92.9)	91.3 (84.2–98.9)	0.194
LFS, probability (95% CI)	76.2 (69.3–83.7)	70.0 (60.1–81.7)	83.7 (74.9–93.5)	0.122
GRFS, probability (95% CI)	67.9 (60.0–76.7)	68.7 (58.3–81.1)	66.3 (54.9–80.0)	0.371
EFS, probability (95% CI)	80.7 (74.1–87.9)	73.6 (63.9–84.9)	89.5 (81.8–97.8)	0.028

aGvHD = acute graft-versus-host disease, cGvHD = chronic graft-versus-host disease, CIs = cumulative incidence, CMV = cytomegalovirus, EBV = Epstein–Barr virus, EFS = event-free survival, GRFS = graft-versus-host disease–relapse-free survival, LFS = leukemia-free survival, m/s cGvHD = moderate to severe chronic graft-versus-host disease, MRD = measurable residual disease, NRM = non-relapse mortality, OS = overall survival.

Patients were classified into 2 groups based on the development of HF: HF and non-HF (NHF) groups. HF occurred in 62 patients (43.1%), including 41 who received PTCy and 21 who received ATG. HF was associated with higher infused counts of mononuclear cells (MNCs) (14.9 vs 12.8 × 10^8^/kg; *p* = 0.026) and CD34^+^ cells (9.8 vs 8.8 × 10^6^/kg; *p* = 0.008). Patients receiving PTCy-based GvHD prophylaxis had a significantly higher incidence of HF compared with those receiving ATG (66.1% vs 33.9%; *p* = 0.001).

### 3.2. Impact of HF on acute and chronic GvHD

**Figure 2.**
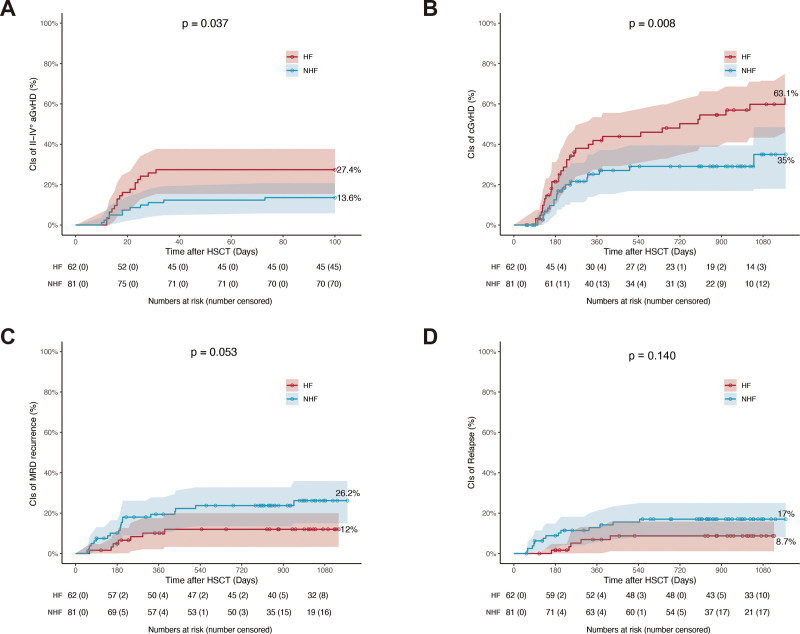
Incidence of transplant complications associated with HF. Cumulative incidence of (A) grade II–IV aGvHD, (B) 3-y cGvHD, (C) 3-y MRD recurrence, and (D) 3-y relapse. aGvHD = acute graft-versus-host disease, CI = confidence interval, HF = haplo-fever, HSCT = hematopoietic stem cell transplantation, MRD = measurable residual disease, NHF = non-HF.

The cumulative incidence of grade II to IV aGvHD was significantly higher in the HF group compared with the NHF group (27.4% [95% CI: 18.0%–40.3%] vs 13.6% [95% CI: 7.8%–23.2%]; *p* = 0.043) (**Fig. 2A**). By contrast, the cumulative incidence of grade III to IV aGvHD was comparable between the 2 groups (Supplemental Figure 1A, https://links.lww.com/BS/A143). The 3-year probability of cGvHD was 63.1% (95% CI: 45.9%–74.9%) in the HF group and 35.0% (95% CI: 17.1%–48.5%) in the NHF group (*p* < 0.005) (**Fig. 2B**, **Table 2**). The cumulative incidences of moderate to severe cGvHD (m/s cGvHD), EBV reactivation, CMV reactivation, and NRM did not differ significantly between the 2 groups (Supplemental Figure 1B–D, https://links.lww.com/BS/A143, **Table 2**).

In univariate analysis, HF was associated with an increased risk of both grade II to IV aGvHD (hazard ratio [HR], 2.2; 95% CI: 1.0–4.7; *p* = 0.042) (Supplemental Table 1, https://links.lww.com/BS/A143) and cGvHD (HR, 2.5; 95% CI: 1.5–4.3; *p* < 0.001) (Supplemental Table 2, https://links.lww.com/BS/A143). Multivariate analysis confirmed HF as an independent risk factor for grade II to IV aGvHD (HR, 2.53; 95% CI: 1.07–5.99; *p* = 0.035) and cGvHD (HR, 3.00; 95% CI: 1.54–5.83; *p* < 0.001) after adjustment for other variables.

### 3.3. Impact of HF on survival outcomes

The cumulative incidence of 3-year probability of MRD recurrence was lower in the HF group compared with the NHF group (12.0% [95% CI: 3.2%–20.0%] vs 26.2% [95% CI: 15.0%–35.9%]; *p* = 0.053) (**Fig. 2C**, **Table 2**). By contrast, the 3-year cumulative incidence of relapse did not differ significantly between the 2 groups (**Fig. 2D**, **Table 2**). The 3-year probability of EFS after allo-HSCT was 89.5% (95% CI: 81.8%–97.8%) in the HF group and 73.6% (95% CI: 63.9%–84.9%) in the NHF group (*p* = 0.028) (Supplemental Figure 2A, https://links.lww.com/BS/A143, **Table 2**). The 3-year probability of OS, LFS, and GRFS were not significantly different between the HF and NHF groups (Supplemental Figure 2B–D, https://links.lww.com/BS/A143, **Table 2**).

Multivariate analysis showed that cGvHD was a protective factor, while pre-transplant MRD positivity was a risk factor for both MRD recurrence and inferior EFS (Supplemental Tables 3–4, https://links.lww.com/BS/A143). To evaluate whether HF influenced MRD recurrence and EFS indirectly through cGvHD, mediation analysis was performed with HF as the independent variable and cGvHD as the mediator. The average causal mediation effect (ACME) values were 2820 (95% CI: 186–12,106; *p* = 0.016) for MRD recurrence and 3920 (95% CI: 237–17,878; *p* = 0.016) for EFS. Approximately 45% of the effect of HF on MRD recurrence and 46% of the effect on EFS were mediated by cGvHD (Supplemental Table 5, https://links.lww.com/BS/A143).

### 3.4. Impact of HF with different GvHD prophylaxis

The overall incidence of HF was significantly lower in patients receiving ATG-based GvHD prophylaxis compared with those receiving PTCy-based prophylaxis (29.6% [21/71] vs 56.9% [41/72]; *p* = 0.001) (**Table 1**). Although previous studies reported that HF in PTCy-based HID HSCT was a risk factor for NRM,^10,11^ 1-year OS, DFS,^11^ and GRFS,^12^ the impact of HF in ATG-based HID HSCT remains less well defined.

**Figure 3.**
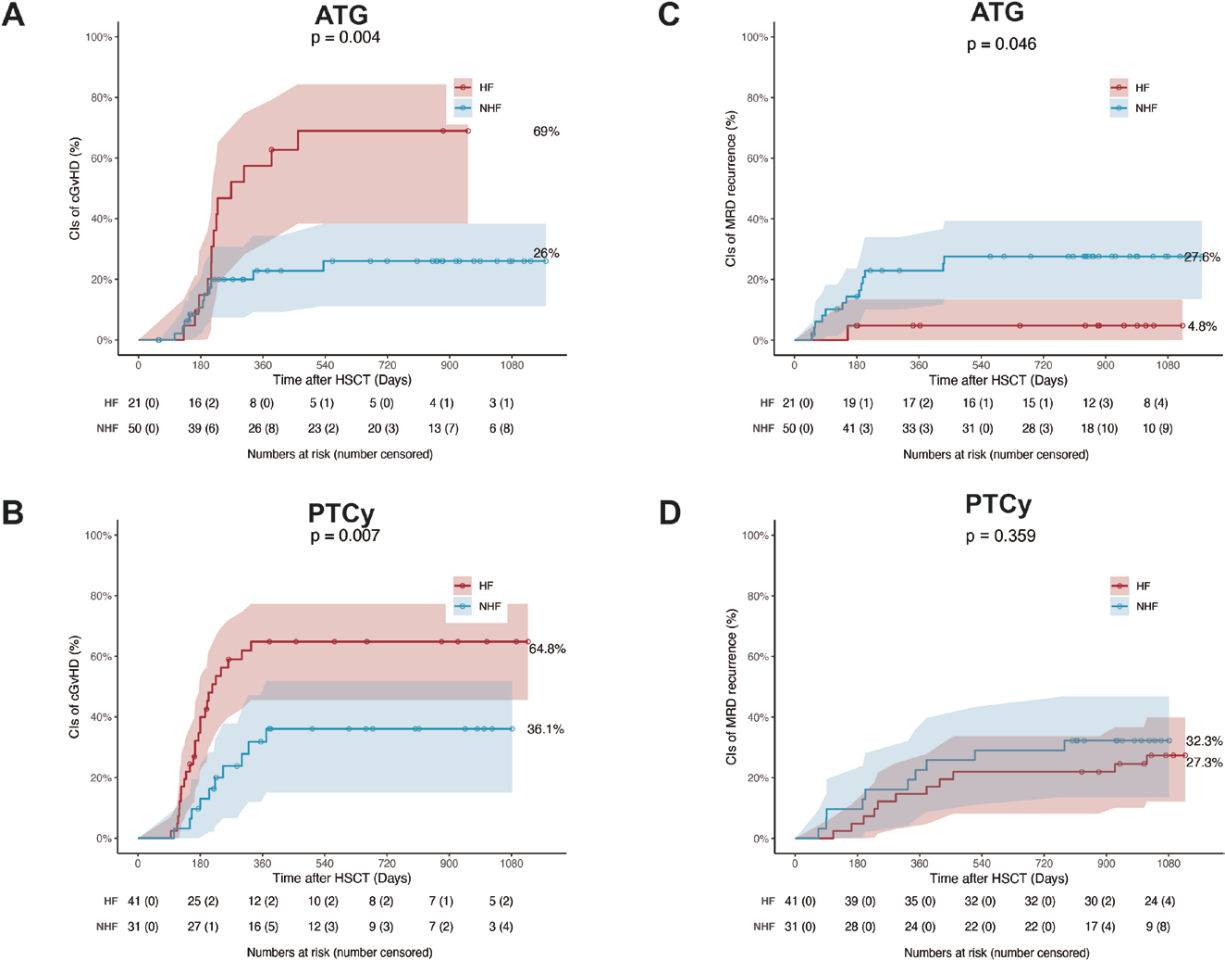
Transplant outcomes in ATG and PTCy cohorts. (A,C) Cumulative incidence of 3-y cGvHD and (B,D) 3-y MRD recurrence in the ATG and PTCy cohorts, respectively. ATG = anti-thymocyte globulin, cGvHD = chronic graft-versus-host disease, CI = confidence interval, HF = haplo-fever, HSCT = hematopoietic stem cell transplantation, MRD = measurable residual disease, NHF = non-HF, PTCy = post-transplantation cyclophosphamide.

In the ATG-based prophylaxis group, patients with HF exhibited a significantly higher cumulative incidence of 3-year probability cGvHD (69.0% [95% CI: 38.4%–74.4%] vs 26.0% [95% CI: 11.2%–38.4%]; *p* = 0.004), a lower incidence of 3-year MRD recurrence (4.8% [95% CI: 0%–13.4%] vs 27.6% [95% CI: 13.5%–39.1%]; *p* = 0.046), and superior 3-year probability of EFS (95.2% [95% CI: 85.2%–100%] vs 72.0% [95% CI: 60.1%–86.2%]; *p* = 0.046) compared with the NHF group (**Fig. 3A–B**, Supplemental Figure 3A and Table 6, https://links.lww.com/BS/A143). By contrast, within the PTCy-based prophylaxis group, patients with HF exhibited a higher cumulative incidence of grade II to IV aGvHD (29.3% [95% CI: 17.8%–45.7%] vs 6.5% [95% CI: 1.7%–23.4%]; *p* = 0.013) and 3-year probability of cGvHD (64.8% [95% CI: 45.6%–77.3%] vs 36.1% [95% CI: 15.0%–51.9%]; *p* = 0.007) compared with the NHF group (**Fig. 3C–D**, Supplemental Figure 3B and Table 7, https://links.lww.com/BS/A143).

### 3.5. Risk factors for HF in ATG-based HID HSCT

**Figure 4.**
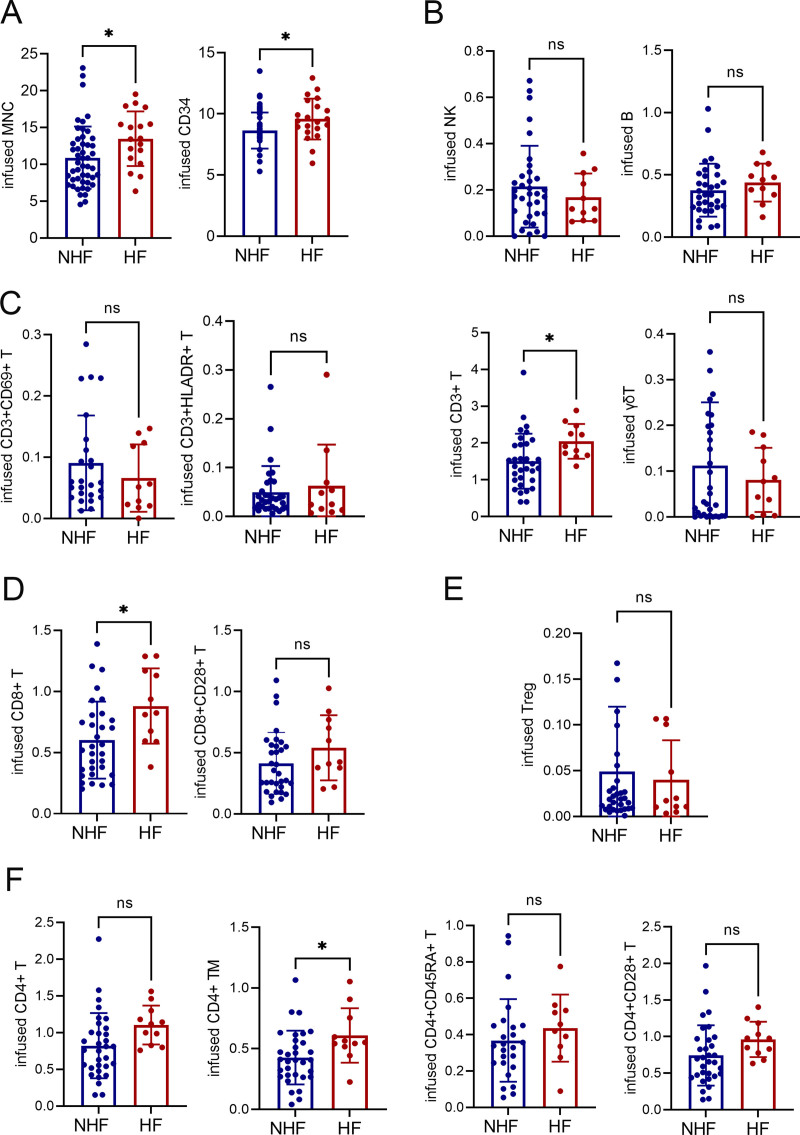
Association between infused cell counts and HF development. (A) Amounts of infused MNCs and CD34^+^ cells; (B) CD3^+^ T cells, NK cells, B cells, and γδ T cells; (C) CD3^+^CD69^+^ and CD3^+^HLADR^+^ T cells; (D) CD8^+^ and CD8^+^CD28^+^ T cells; (E) Treg cells; and (F) CD4^+^, CD4^+^ TM, CD4^+^CD45RA^+^, and CD4^+^CD28^+^ T cells in the NHF and HF groups. TM, memory T cells. *p < 0.050. HF = haplo-fever, MNC = mononuclear cell, NHF = non-HF, NK = natural killer.

In the PTCy setting, established risk factors for HF include the infused doses of CD34^+^ cells, MNCs, and CD3^+^ T cells^4,11^; however, risk factors for HF in the ATG setting have not been well investigated. In our study, baseline characteristics between patients with HF and those without HF (NHF) in the ATG cohort were comparable (Supplemental Table 8, https://links.lww.com/BS/A143). Patients in the HF group had significantly higher infused doses of CD34^+^ cells (9.6 vs 8.6 × 10^6^/kg; *p* = 0.020), MNCs (13.5 vs 10.9 × 10^9^/kg; *p* = 0.026), CD3^+^ T cells (2.0 vs 1.5 × 10^8^/kg; *p* = 0.030), and CD8^+^ T cells (0.9 vs 0.6 × 10^8^/kg; *p* = 0.015) compared with the NHF group (**Fig. 4A–F**).

Univariate logistic regression further identified the infused amounts of CD3^+^ T cells, CD4^+^ T cells, and CD8^+^ T cells as risk factors for HF (Supplemental Table 9, https://links.lww.com/BS/A143). These findings suggest that the risk factors for HF in the ATG cohort are similar to those reported in the PTCy setting.^4,11^ Moreover, analysis of lymphocyte subsets revealed that the infused dose of CD4^+^ memory T (TM) cells was significantly higher in the HF group compared with the NHF group (0.6 vs 0.4 × 10^8^/kg; *p* = 0.024) (**Fig. 4F**, Supplemental Table 9, https://links.lww.com/BS/A143).

### 3.6. Impact of HF on immune reconstitution in patients with ATG-based aGvHD prophylaxis

Immune reconstitution is closely associated with the risks of GvHD, relapse, infections, and survival.^27,28^ Although we found that HF was linked to aGvHD, cGvHD, MRD recurrence, and EFS, its impact on immune reconstitution is unknown. We therefore delineated the profile of immune reconstitution in patients with and without HF who received ATG-based aGvHD prophylaxis.

**Figure 5.**
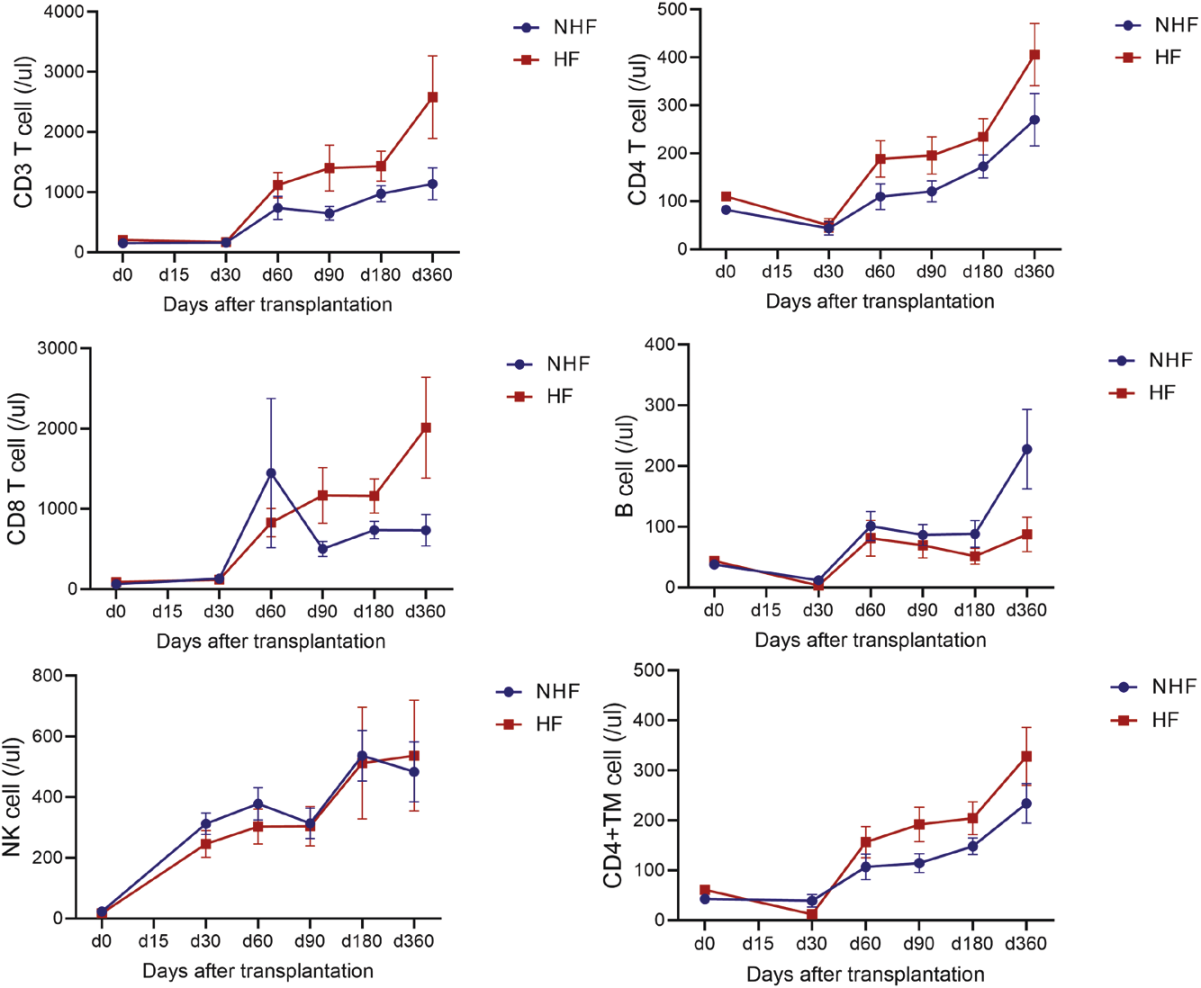
The recover trajectory of lymphocyte subsets in NHF and HF groups. Absolute counts of peripheral blood lymphocyte subsets, including CD3^+^ T cells, CD4^+^ T cells, CD8^+^ T cells, B cells, NK cells, and CD4^+^ TM cells, were longitudinally monitored at indicated time points (d0 to d360) for the HF group and NHF group. Data are presented as mean ± SEM. Statistical significance was determined using a mixed-effects model, treating time post-transplantation and group as fixed factors to accommodate the longitudinal nature of the data and potential missing values. Pgroup and Ptime indicate the overall main effects of the cohort and follow-up duration, respectively. ATG = anti-thymocyte globulin, HF = haplo-fever, HID = haploidentical, HSCT = hematopoietic stem cell transplantation, NHF = non-HF, NK = natural killer.

First, we characterized the kinetics of global lymphocyte reconstitution. In the HF group, the absolute numbers of CD3^+^ T cells and CD4^+^ T cells were rapidly reconstituted, with dominance of CD4^+^ TM cells, while B-cell reconstitution was slower compared with the NHF group (**Fig. 5**). Next, we compared the absolute numbers of lymphocyte subsets at 90 days after allo-HSCT to explore how HF might influence cGvHD and MRD recurrence through patterns of immune reconstitution.

Patients with HF showed significantly higher absolute counts of CD3^+^ T cells (1400 vs 647/μL; *p* = 0.019) and CD3^+^CD8^+^ T cells (1168 vs 500/μL; *p* = 0.018) (Supplemental Figure 4A, https://links.lww.com/BS/A143). The absolute number of CD4^+^ TM cells at day 90 was also significantly higher in the HF group compared with the NHF group (192 vs 114/μL; *p* = 0.038). Moreover, T cells from HF patients exhibited greater activation, as evidenced by increased CD28 expression on T-cell populations (CD4^+^CD28^+^: 164 vs 92/μL, *p* = 0.049; CD8^+^CD28^+^: 502 vs 207/μL, *p* = 0.022) (Supplemental Figure 4B–C, https://links.lww.com/BS/A143).

### 3.7. Molecular profiling highlights the impact of activated CD4^+^ TM cells on HF

**Figure 6.**
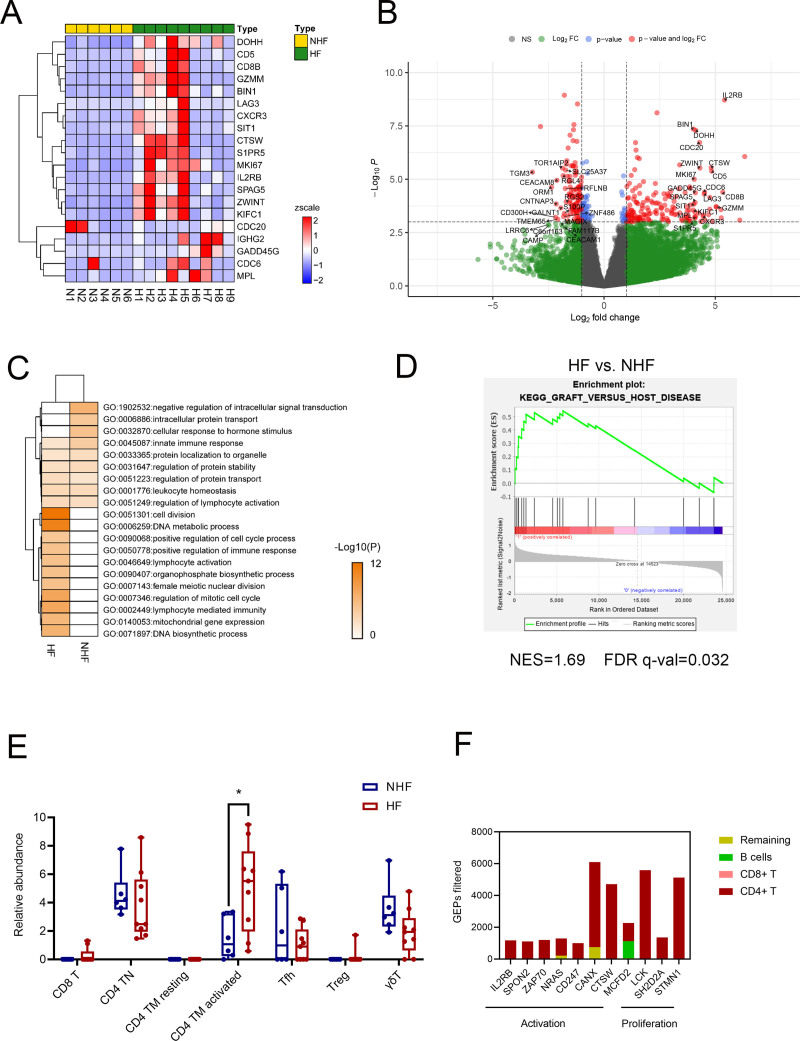
RNA-seq analysis of T cells from NHF and HF groups at day 2 post–HID HSCT with ATG. (A) Heatmap of the top 20 upregulated DEGs in HF vs NHF groups. (B) Volcano plot of DEGs between NHF and HF groups. (C) GO enrichment of upregulated DEGs in HF vs NHF. (D) GSEA of T-cell transcriptomes in HF vs NHF. (E) Abundance of T-cell subsets estimated using CIBERSORTx. (F) Average expression of functional T-cell genes estimated by CIBERSORTx in each T-cell subset. *p < 0.050. ATG = anti-thymocyte globulin, DEGs = differentially expressed genes, FDR = false discovery rate, GEP = gene expression programming, GO = Gene Ontology, GSEA = Gene set enrichment analysis, HF = haplo-fever, HID = haploidentical, HSCT = hematopoietic stem cell transplantation, NES = normalized enrichment score, NHF = non-HF.

To evaluate T-cell function in the development of HF, we performed RNA sequencing of circulating CD3^+^ T cells collected on day 2 after allo-HSCT. Given the very low numbers of residual T cells and minimal RNA yield, we employed the Smart-seq approach, known for its robustness and reliability, to generate libraries for bulk RNA sequencing. Compared with samples from the NHF group, 202 genes were upregulated and 219 genes were downregulated in the HF group (**Fig. 6A–B**, Supplemental Figure 5A, https://links.lww.com/BS/A143). Among the top 20 upregulated genes, many were related to lymphocyte activation (*IL2*R*B*, *CD8B*, *CD5*), differentiation (*DOHH*), cytolytic activity (*GZMM*), cell cycle progression (*CDC6*, *ZWINT*, *MKI67*), and chemotaxis (*CXCR3*, *S1PR5*) (**Fig. 6A–B**).

GO analysis, GSEA, and protein–protein interaction (PPI) network analyses demonstrated that the most upregulated genes were enriched in pathways related to the cell cycle, lymphocyte-mediated immunity, T-cell activation, and GvHD (**Fig. 6C–D**, Supplemental Figure 5B–C, https://links.lww.com/BS/A143). To assess the lymphocyte subsets contributing to HF, we estimated the relative abundance of 7 T-cell subsets using CIBERSORTx. An increased abundance of activated CD4^+^ TM cells was observed in the HF group compared with the NHF group (**Fig. 6E**).

Further analysis showed that genes associated with immune activation (*IL2RB, SPON2, ZAP70*, *NRAS*, *CD247*, *CANX*, *CTSW*) and cell cycle regulation (*LCK*, *SH2D2A*, *STMN1*) were predominantly expressed in CD4^+^ T cells (**Fig. 6F**), suggesting that highly activated and proliferative CD4^+^ TM cells play a major role in the development of HF.

## 4. DISCUSSION

Recent studies have revealed associations between early HF and transplant outcomes, as well as risk factors for HF, in PTCy-based HID HSCT.^4–6,10,12,29^ The incidence and clinical significance of HF in ATG-based adult HID HSCT, however, remain rarely characterized. A previous study reported that the incidence of HF was 38.0% in pediatric patients receiving HID HSCT with ATG.^7^ Consistent with this, we found that the incidence of HF in adult patients receiving ATG (29.6%) was lower than in those receiving PTCy-based HID HSCT (56.9%; *p* = 0.001). Risk factors for HF included higher numbers of infused CD34^+^ cells, MNCs, and T cells, which were similar to those reported in the PTCy setting.^4,11^ Activation of T cells and other proinflammatory cells has been implicated in HF development after PTCy-based HID HSCT.^5^ The lower incidence of HF in the ATG group may be attributable to the administration of ATG on days −4 to −2 before graft infusion, resulting in depletion of pathogenic alloreactive T cells in a T-cell–replete graft. Likewise, other studies have reported that using a combination of ATG and PTCy for GVHD prophylaxis can lower the incidence CRS, without increasing relapse.^30,31^ In a recent study by Storek et al,^29^ HF occurred in 44% patients who received 3 infusions of ATG on day −2, −1, and 0, the levels of several cytokines, including IL-1RA and IL-6, were significantly elevated compared to those without HF.

Although HF patients have a higher risk of GvHD, they also demonstrate lower MRD positivity and superior EFS indicating an underestimated association with an enhanced graft-versus-leukemia (GvL) effect. Causal mediation analysis indicated that HF had an indirect effect on MRD recurrence and EFS, partially mediated by cGvHD, indicating that additional factors may also be involved. Its pathogenesis and immune reconstitution patterns vary with GvHD prophylaxis; in the ATG group, HF is linked to a higher dose of infused CD4^+^ memory T cells, driving rapid immune reconstitution dominated by highly activated CD4^+^ T cells, it may serve as a prognostic marker identifying individuals with enhanced GvL activity.^32^Thus, HF identifies a favorable patient subset with active immunity and an enhanced GvL effect, holding promise for clinical translation. In murine models, GvL effects are driven by CD4^+^ TM cells that require cognate interactions with MHC class II and leukemia antigens.^33^ Infusion of CD4^+^ TM cells into recipients of T cell–depleted human allografts has also been shown to enhance GvL.^34^ The association between HF and rapid recovery of CD4^+^ TM cells suggests that these cells might mediate the effect of HF on MRD recurrence and EFS. Future prospective studies are warranted to comprehensively assess its predictive value in transplant outcomes.

Through magnetic bead sorting combined with the Smart-seq method, we performed RNA sequencing analysis on T cells that were significantly reduced in number following ATG treatment, providing preliminary clues for the molecular mechanisms of HF. The low-input RNA sequencing approach employed in this study may serve as a reference for subsequent molecular investigations involving similar limited samples. We then employed CIBERSORTx to estimate immune cell profiles from RNA expression data. Molecular profiling of CD3^+^ T cells revealed transcriptomic signatures of T-cell proliferation and activation during HF development. The presence of activated CD4^+^ TM cells at day 2 post-transplant suggests that this population might be resistant to ATG-mediated depletion. This finding is consistent with multiple studies demonstrating that a subset of memory T cells survives lymphoablative induction with ATG.^35–37^ Furthermore, expansion of depletion-resistant memory T cells has been associated with acute rejection in renal transplantation.^38^ ATG depletes T lymphocytes via complement-dependent lysis and apoptosis, with lower concentrations (10 mg/mL) sufficient to eliminate activated T cells compared to those required for resting T cells (100 mg/mL).^39^ The resting state and longevity of memory T cells may confer their survival advantage against ATG clearance.

The higher expression of IL2RB in the HF group aligns with the role of IL2–IL2RB signaling in enhancing the cytotoxic responses of effector T cells, memory T cells, and natural killer cells, without promoting immune tolerance via regulatory T cells.^40,41^ In ATG-based HID HSCT, the high frequency of CD4^+^ TM cells in the graft, the abundance of activated CD4^+^ TM cells at day 2, and the rapid reconstitution of CD4^+^ TM cells by 3 months underscore the critical role of persistent CD4^+^ TM cells in the pathogenesis and clinical outcomes of HF. Further study into the specific mechanisms of the major cell subset mediating HF, CD4^+^ TM cells, may enhance our understanding of GvL effects. Such insights could guide strategies to optimize the drug administration or adjust the composition of cell populations in the graft, with the goal of enhancing the function of this cell subset or activating related pathways to improve patient outcomes.

Several limitations of our study should be acknowledged. These include its retrospective single-center design, relatively small sample size, and short follow-up period. Although we followed patients with a median time of 1001 days, the limited number of enrolled patients also restricted the evaluation of more detailed immune cell subpopulations that may mediate the effects of HF on MRD recurrence and EFS. Although we assessed depletion-resistant T-cell subsets and enriched pathways contributing to HF through in silico analyses, further studies are needed to confirm the roles of specific T-cell subsets and other immune cells, such as antigen-presenting cells, in HF development.

In conclusion, our data highlight the impact of HF on transplant outcomes, identify risk factors for HF, and elucidate molecular mechanisms of T-cell activation involved in HF among HID HSCT recipients receiving ATG-based GvHD prophylaxis. HF was associated with a higher dose of infused CD4^+^ TM cells, persistence after ATG administration, and rapid reconstitution of CD4^+^ TM cells. Overall, HF might serve as a predictor of cGvHD in the absence of established biomarkers for allogenic immune complications.

## ACKNOWLEDGMENTS

This study was supported by grants from the National Science and Technology Major Project (2025ZD0545700 to X.H.), the National Natural Science Foundation of China (82170206 to X.H. and 82200156 to Z.P.), the Shanghai Municipal Health Commission (Project of Disciplines of Excellence 20234Z0002 to X.H., and Project of Clinical Research in Health 20214Y0187 to Z.P.), and the Science and Technology Commission of Shanghai Municipality (Project of Medical Innovation Research 23Y11904500 to Z.P.).

## ETHICAL APPROVAL

The study was approved by the institutional review board of Ruijin Hospital (approval number: 2021-388) and conducted in accordance with the *Declaration of Helsinki*.

## AUTHOR CONTRIBUTIONS

Z.P., H.H., and T.L. processed patient samples, collected clinical data, analyzed RNA-seq data, and wrote the manuscript. H.D. and Q.G. developed the methodology. J.H. and H.H. acquired the clinical data. X.H., Z.P., and H.C. proposed and designed the study, interpreted the data, wrote the manuscript, and oversaw the project. All authors read and approved the final manuscript.

## Supplementary Material


