# Supplementary material for: Anti-thymocyte globulin-resistant CD4+ memory T cells contribute to haplo-fever after allogeneic hematopoietic stem cell transplantation
Source: Blood Sci. 2026 Feb 26;8(1):e00280. doi: 10.1097/BS9.0000000000000280 (PMC12947993; doi:10.1097/BS9.0000000000000280)
Supplement: Supplementary file 1 [file bs9-8-e00280-s001.pdf]

Supplemental Appendix

Anti-thymocyte globulin resistant CD4$^+$ memory T cells contribute to haplo-fever after allogeneic hematopoietic stem cell transplantation

Supplemental Figures

Supplemental Fig.1 Incidence of transplant complications in association with HF........................2
Supplemental Fig.2 Survival outcomes in association with HF...3
Supplemental Fig.3 EFS and aGvHD in ATG and PTCy cohorts...4
Supplemental Fig.4 Immune reconstitution at 3 months after allo-HSCT......................................5
Supplemental Fig.5 Expression profile of T cells in NHF and HF groups at 2d after HID-HSCT...6

Supplemental Tables

Supplemental Table 1. Risk factors of grades II-IV aGvHD...7
Supplemental Table 2. Risk factors for cGvHD..8
Supplemental Table 3. Risk factors for MRD recurrence 9
Supplemental Table 4. Risk factors for EFS 10
Supplemental Table 5. Mediation analysis of HF (independent variable) and cGvHD (mediator). 11
Supplemental Table 6. Transplant outcomes of ATG cohort..12
Supplemental Table 7. Transplant outcomes of PTCy cohort..13
Supplemental Table 8. Baseline characteristics of patients in NHF and HF groups in the ATG cohort 14
Supplemental Table 9. Risk factors of HF in ATG cohort..15

Supplemental Figure 1

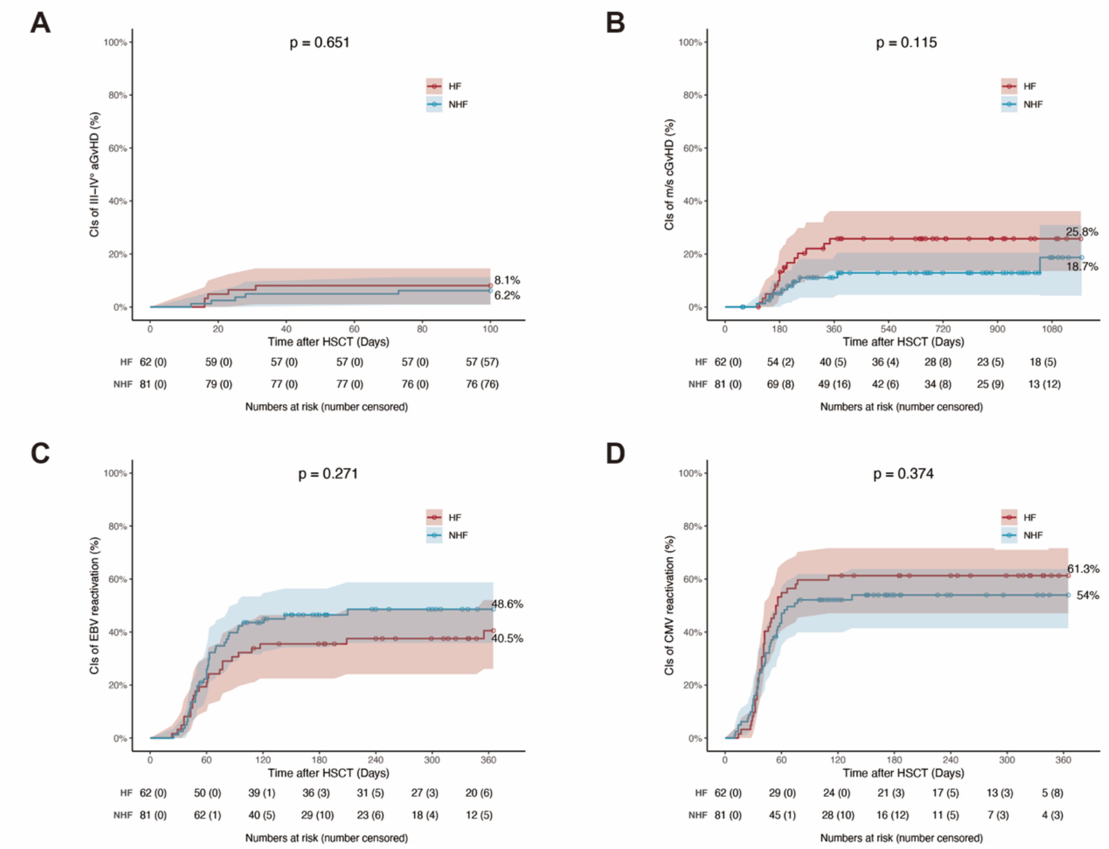

Supplemental Figure 1. Incidence of transplant complications associated with HF. Cumulative incidence of grade III–IV aGvHD (A), 3-year moderate to severe cGvHD (B), 1-year CMV reactivation (C), and 1-year EBV reactivation (D). m/s cGvHD, moderate to severe chronic GvHD.

Supplemental Figure 2

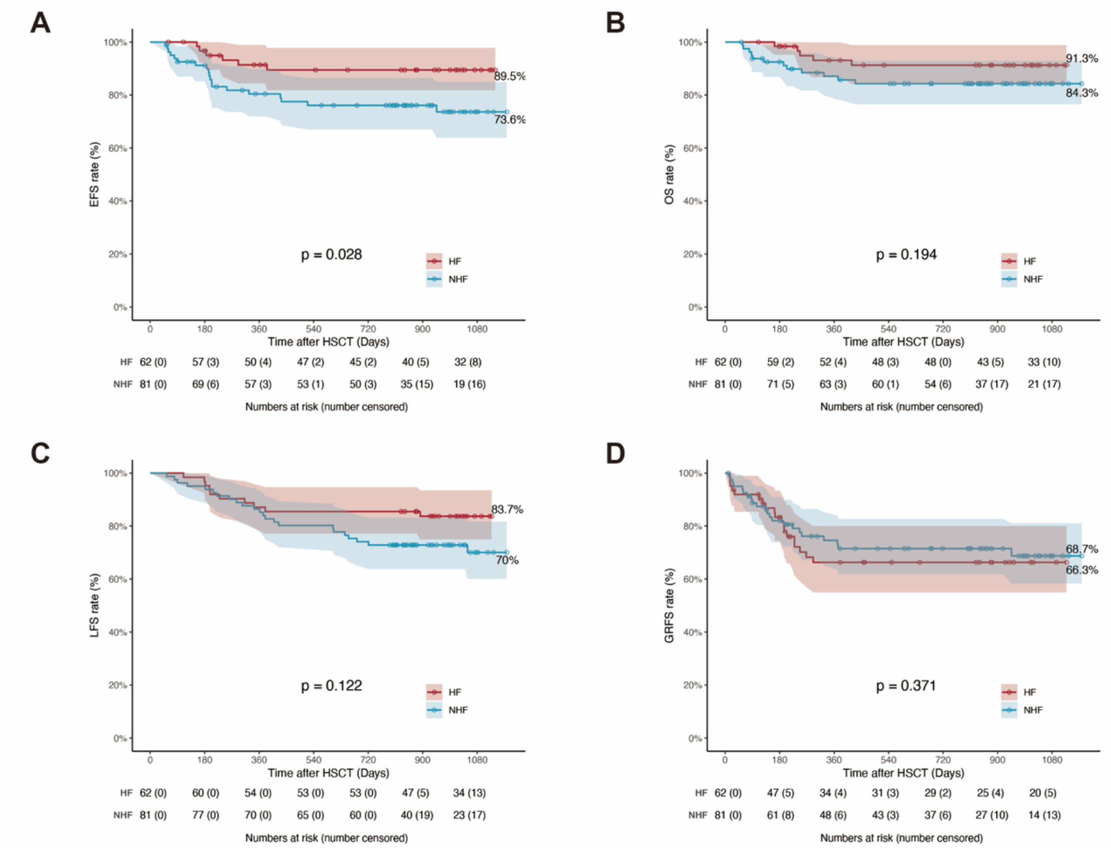

Supplemental Figure 2. Survival outcomes associated with HF. 3-year probability of event-free survival (EFS) (A), overall survival (OS) (B), leukemia-free survival (LFS) (C), and graft-versus-host disease–relapse–free survival (GRFS) (D).

Supplemental Figure 3

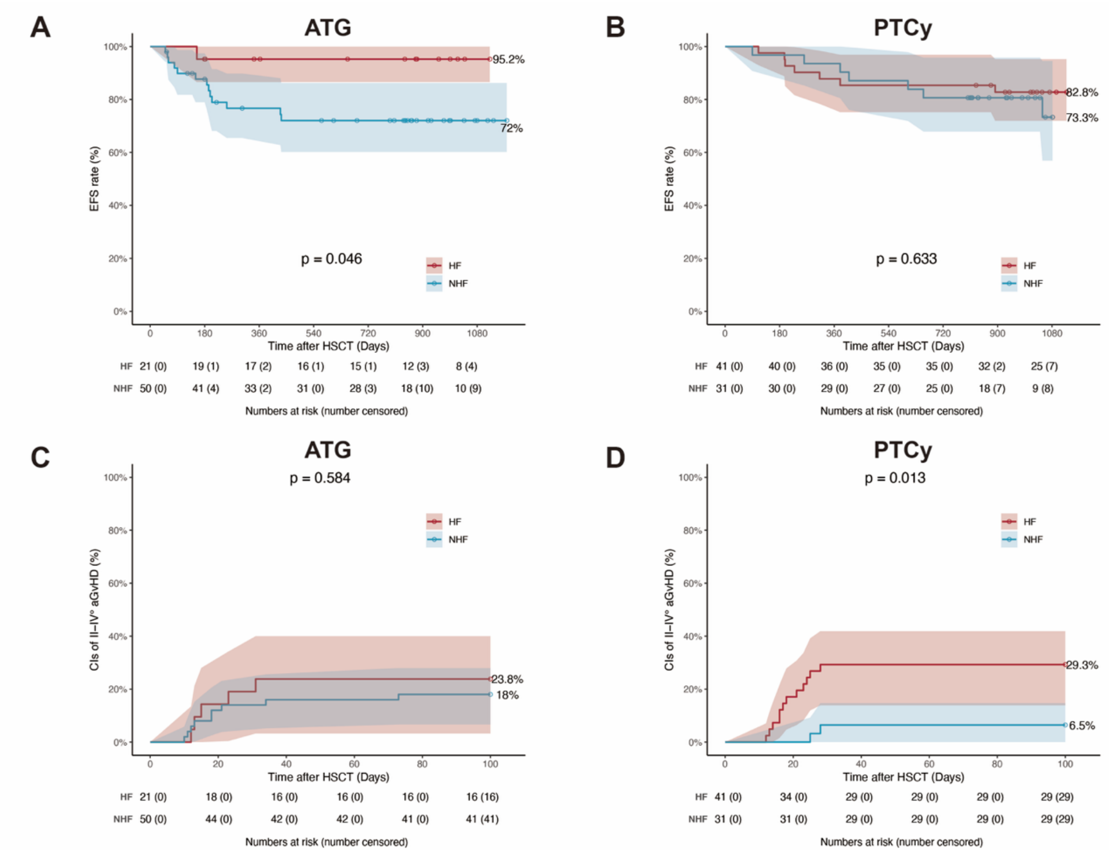

Supplemental Figure 3. EFS and aGvHD in ATG and PTCy cohorts. 3-year probability of event-free survival (EFS) (A) and cumulative incidence of grade II–IV aGvHD (B).

Supplemental Figure 4

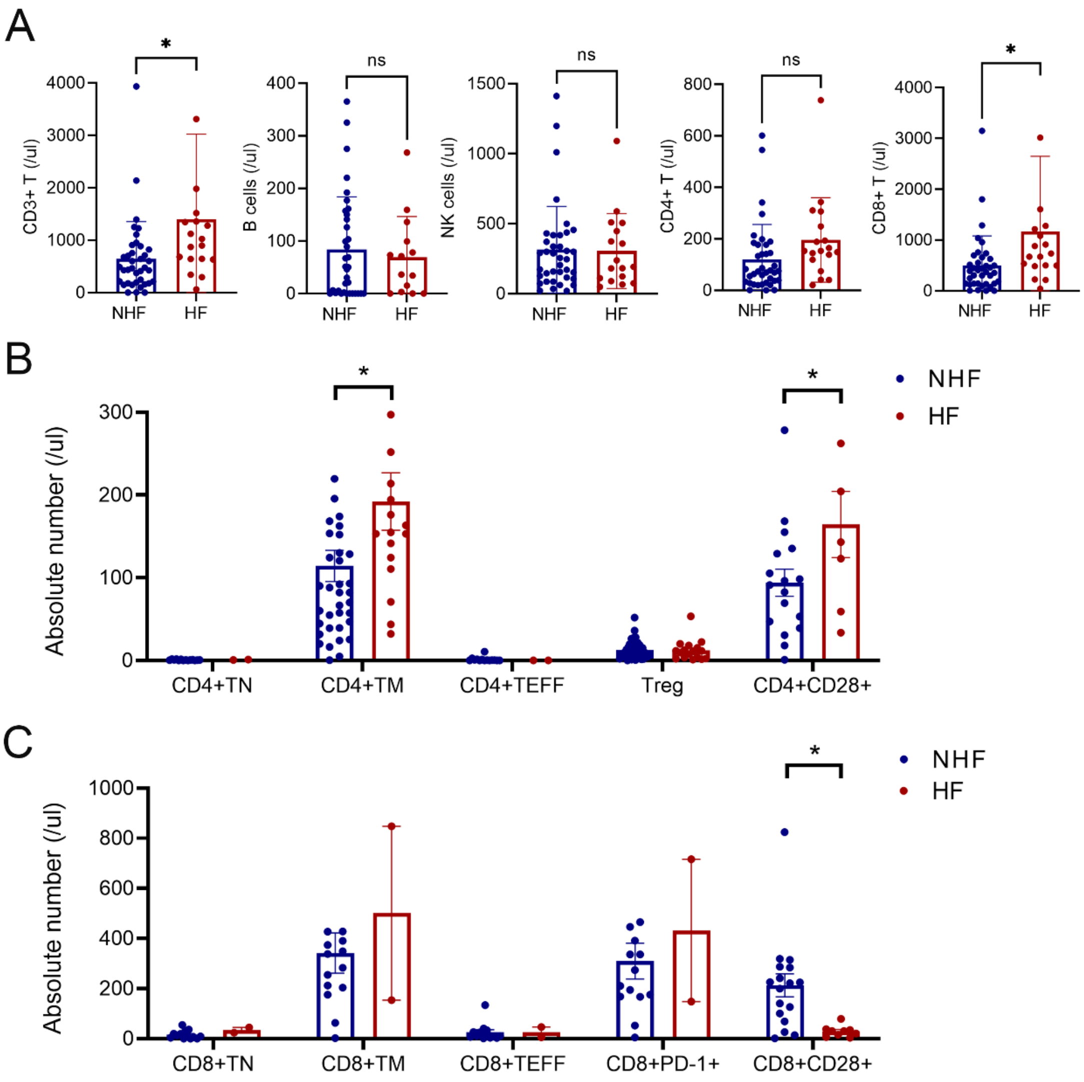

Supplemental Figure 4. Immune reconstitution at 3 months after allo-HSCT. Absolute numbers of $CD3^{+}$ T cells, B cells, NK cells, $CD4^{+}$ T cells, and $CD8^{+}$ T cells in NHF and HF groups (A). Absolute numbers of $CD4^{+}$ T-cell subsets (B) and $CD8^{+}$ T-cell subsets (C) in NHF and HF groups. $P < 0.05$.

Supplemental Figure 5

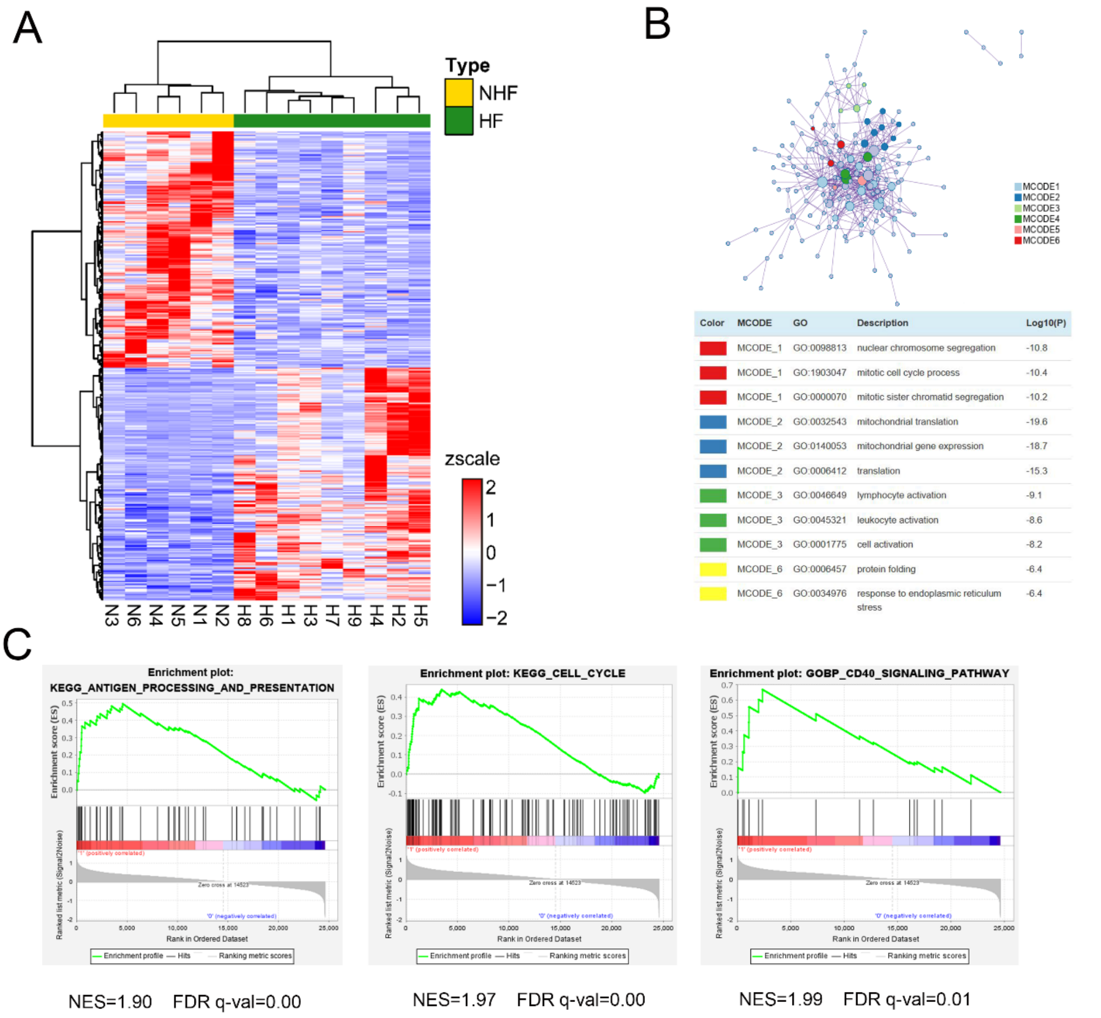

Supplemental Figure 5. Expression profiles of T cells in NHF and HF groups at day 2 after HID-HSCT. Heatmap of differentially expressed genes (DEGs) in NHF and HF groups (A). Protein-protein interaction (PPI) network of DEGs analyzed using Metascape and MCODE algorithm (B). Representative gene set enrichment analysis (GSEA) of T-cell transcriptomes in HF vs. NHF groups (C). DEGs, differentially expressed genes.

Supplemental Table 1. Risk factors of grades II-IV aGvHD

Risk factors	Univariate Cox		Multivariate Cox	
	HR (95% CI)	*P* value	HR (95% CI)	*P* value
Categorical variables				
**HF**	2.2(1-4.7)	0.04	2.53 (1.07-5.99)	0.03
**CMV_reactivation**	1.7(0.78-3.8)	0.17	2.27 (0.87-5.93)	0.10
**Gender match**				
Female_to_Male	Ref	Ref	Ref	Ref
Others	0.67(0.29-1.6)	0.37	0.76 (0.29-1.94)	0.56
**Pre_conditioning**				
MAC	Ref	Ref	Ref	Ref
RIC	1.1(0.27-4.8)	0.85	0.61 (0.07-5.08)	0.65
**Diagnosis**				
Myeloid	0.79(0.37-1.7)	0.55	0.46 (0.17-1.2)	0.11
Lymphoid	Ref	Ref	Ref	Ref
**Disease_status**				
Remission	1.1(0.39-3.2)	0.84	1.26 (0.34-4.71)	0.73
Active	Ref	Ref	Ref	Ref
**GvHD_prophylaxis**				
PTCy	0.96(0.46-2)	0.92	0.61 (0.26-1.45)	0.26
ATG	Ref	Ref	Ref	Ref
Continuous variables				
**Infused CD34 cells**	1.1(0.9-1.3)	0.45	1.04 (0.85-1.26)	0.70
**Infused MNCs**	1.1(1-1.2)	0.06	1.08 (0.99-1.17)	0.09
**Age**	1(0.98-1)	0.50	1.02 (0.99-1.06)	0.14

Abbreviations: HF, haplo-fever; aGvHD, acute graft-versus-host disease; CMV, cytomegalovirus; MAC, myeloablative conditioning; RIC, reduced-intensity conditioning; MNC, mononuclear cells; $CD34^+$, cluster of differentiation 34 positive.

Supplemental Table 2. Risk factors for cGvHD.

Risk factors	Univariate Cox		Multivariate Cox	
	HR (95% CI)	*P* value	HR (95% CI)	*P* value
Categorical variables				
**HF**	2.5(1.5-4.3)	0.00	3 (1.54-5.83)	0.00
**CMV_reactivation**	1.8(1-3.2)	0.04	1.52 (0.77-3.03)	0.23
**Grades II-IV aGvHD**	1.3(0.68-2.3)	0.45	1.12 (0.55-2.3)	0.75
**DLI**	1.2(0.44-3.4)	0.70	2 (0.62-6.51)	0.25
**Gender match**				
Female_to_Male	Ref	Ref	Ref	Ref
Others	0.74(0.4-1.3)	0.32	0.58 (0.29-1.12)	0.10
**Pre_conditioning**				
MAC	Ref	Ref	Ref	Ref
RIC	0.66(0.21-2.1)	0.48	0.36 (0.08-1.59)	0.18
**Diagnosis**				
Myeloid	1.9(1-3.5)	0.04	2.68 (1.31-5.47)	0.01
Lymphoid	Ref	Ref	Ref	Ref
**Disease_status**				
Remission	1.5(0.66-3.6)	0.32	2 (0.79-5.04)	0.14
Active	Ref	Ref	Ref	Ref
**GvHD_prophylaxis**				
PTCy	0.95(0.43-2.1)	0.90	0.66 (0.27-1.64)	0.37
ATG				
Continuous variables				
**Infused CD34 cells**	1.1(0.94-1.2)	0.28	0.97 (0.83-1.13)	0.72
**Infused MNCs**	1(0.96-1.1)	0.67	1 (0.94-1.07)	0.90
**Age**	0.99(0.97-1)	0.38	1 (0.97-1.02)	0.76

Abbreviations: HF, haplo-fever; cGvHD, chronic graft-versus-host disease; aGvHD, acute graft-versus-host disease; CMV, cytomegalovirus; DLI, donor lymphocyte infusion; MAC, myeloablative conditioning; RIC, reduced-intensity conditioning; MNC, mononuclear cells; $CD34^+$, cluster of differentiation 34 positive.

Supplemental Table 3. Risk factors for MRD recurrence.

**Risk factors**	**Univariate Cox**		**Multivariate Cox**	
	**HR (95% CI)**	***P* value**	**HR (95% CI)**	***P* value**
Categorical variables				
**HF**	0.37(0.15-0.88)	0.02	0.51 (0.15-1.81)	0.30
**CMV_reactivation**	0.38(0.17-0.85)	0.02	0.47 (0.17-1.31)	0.15
**Grades II-IV aGvHD**	0.3(0.072-1.3)	0.11	0.14 (0.02-1.28)	0.08
**cGvHD**	0.22(0.081-0.58)	0.00	0.23 (0.06-0.84)	0.03
**DLI**	1.2(0.44-3.4)	0.70	2 (0.62-6.51)	0.25
**pre_HCT_MRD**				
positive	4.2(1.8-10)	0.00	7.81 (2.65-23.02)	0.00
negative	Ref	Ref	Ref	Ref
**Gender match**				
Female_to_Male	Ref	Ref	Ref	Ref
Others	1.3(0.46-3.9)	0.58	0.86 (0.23-3.18)	0.82
**Pre_conditioning**				
MAC	Ref	Ref	Ref	Ref
RIC	2.3(0.68-7.6)	0.18	0.87 (0.16-4.67)	0.87
**Diagnosis**				
Myeloid	0.85(0.38-1.9)	0.69	0.38 (0.13-1.13)	0.08
Lymphoid	Ref	Ref	Ref	Ref
**Disease_status**				
Remission	0.76(0.26-2.2)	0.62	3.57 (0.79-16.06)	0.10
Active	Ref	Ref	Ref	Ref
**GvHD_prophylaxis**				
PTCy	0.72(0.33-1.6)	0.41	1.02 (0.38-2.7)	0.97
ATG	Ref	Ref	Ref	Ref
Continuous variables				
**Infused CD34 cells**	0.98(0.81-1.2)	0.83	1.15 (0.9-1.46)	0.27
**Infused MNCs**	0.93(0.84-1)	0.12	0.95 (0.84-1.06)	0.36
**Age**	1(0.99-1.1)	0.21	1.01 (0.98-1.05)	0.39

Abbreviations: HF, haplo-fever; MRD, measurable residual disease; CMV, cytomegalovirus; aGvHD, acute graft-versus-host disease; cGvHD, chronic graft-versus-host disease; DLI, donor lymphocyte infusion; pre_HCT-MRD, MRD status before transplantation; MAC, myeloablative conditioning; RIC, reduced-intensity conditioning; MNC, mononuclear cells; $CD34^+$, cluster of differentiation 34 positive.

Supplemental Table 4. Risk factors for EFS.

Risk factors	Univariate Cox		Multivariate Cox	
	HR (95% CI)	*P* value	HR (95% CI)	*P* value
Categorical variables				
**HF**	0.31(0.12-0.78)	0.01	0.53 (0.16-1.76)	0.30
**CMV_reactivation**	0.36(0.15-0.81)	0.02	0.54 (0.2-1.49)	0.24
**Grades II-IV aGvHD**	0.32(0.076-1.4)	0.12	0.12 (0.01-1.07)	0.06
**cGvHD**	0.23(0.086-0.62)	0.00	0.14 (0.03-0.57)	0.01
**pre_HCT_MRD**				
positive	4.0 (1.7-9.5)	0.00	8 (2.76-23.21)	0.00
negative	Ref	Ref	Ref	Ref
**Gender match**				
Female_to_Male	Ref	Ref	Ref	Ref
Others	1.3(0.44-3.7)	0.66	0.58 (0.17-2.06)	0.40
**Pre_conditioning**				
MAC	Ref	Ref	Ref	Ref
RIC	2.4(0.71-7.9)	0.16	0.58 (0.11-3.01)	0.51
**Diagnosis**				
Myeloid	0.97(0.42-2.2)	0.94	0.68 (0.24-1.92)	0.47
Lymphoid	Ref	Ref	Ref	Ref
**Disease_status**				
Remission	0.73(0.25-2.1)	0.57	4.21 (1.02-17.45)	0.05
Active	Ref	Ref	Ref	Ref
**GvHD_prophylaxis**				
PTCy	0.33(0.045-2.4)	0.28	0.14 (0.02-1.34)	0.09
ATG	Ref	Ref	Ref	Ref
Continuous variables				
**Infused CD34 cells**	1(0.82-1.2)	0.96	1.31 (1-1.71)	0.05
**Infused MNCs**	0.93(0.85-1)	0.13	0.97 (0.87-1.07)	0.53
**Age**	1(0.99-1.1)	0.21	1.01 (0.98-1.04)	0.61

Abbreviations: HF, haplo-fever; EFS, event-free survival; CMV, cytomegalovirus; aGvHD, acute graft-versus-host disease; cGvHD, chronic graft-versus-host disease; pre_HCT-MRD, measurable residual disease status before transplantation; MAC, myeloablative conditioning; RIC, reduced-intensity conditioning; MNC, mononuclear cells; $CD34^+$, cluster of differentiation 34 positive.

Supplemental Table 5. Mediation analysis of HF (independent variable) and cGvHD (mediator).

Outcome	ACME	*P*-value	Prop.Mediated	*P*-value
	coefficient (95% CI)		coefficient (95% CI)	
MRD recurrence	2820(186-12106)	0.02	0.45 (-0.09-2.09)	0.06
EFS	3920 (237-17878)	0.02	0.46 (0.03-1.9)	0.04

Abbreviations: HF, haplo-fever; cGvHD, chronic graft-versus-host disease; MRD, measurable residual disease; EFS, event-free survival; ACME, average causal mediation effects; Prop. Mediated, proportion of the effect mediated through the mediator.

Supplemental Table 6. Transplant outcomes of ATG cohort.

**Transplant outcomes**	**ATG cohort (n=71)**	**NHF group (n=50)**	**HF group (n=21)**	***P* value**
**CIs of aGvHD, incidence (95% CI)**				
I-IV°	31.1 (21.7-43.2)	28.2 (17.8-43.0)	38.1 (21.2-61.9)	0.84
II-IV°	19.7 (12.2-31.0)	18.0 (9.8-31.7)	23.8 (10.7-48.1)	0.58
III-IV°	7.0 (3.8-16.1)	6.2 (2.6-14.2)	8.1 (3.5-18.2)	0.61
**CIs of cGvHD, incidence (95% CI)**				
Total cGvHD	40.1 (25.9-51.6)	26.0 (11.2-38.4)	69.0 (38.4-74.4)	0.004
m/s cGvHD	12.9 (4.1-20.9)	8.8 (0.2-16.7)	21.1 (0.4-37.4)	0.258
**CIs of CMV reactivation, incidence (95% CI)**	51.6 (40.5-63.8)	49.2 (36.1-64.1)	57.1 (37.6-78.1)	0.66
**CIs of EBV reactivation, incidence (95% CI)**	53.4 (41.7-66.1)	55.2 (41.0-70.4)	48.6 (29.6-71.6)	0.43
**CIs of Relapse, incidence (95% CI)**	16.8 (7.2-25.4)	21.6 (8.7-32.6)	4.3 (0-12.8)	0.106
**CIs of MRD recurrence, incidence (95% CI)**	20.9 (10.5-30.2)	27.6 (13.5-39.1)	4.8 (0-13.4)	0.046
**NRM, incidence (95% CI)**	10.4 (2.8–17.5)	12.9 (2.7-22.1)	4.8 (0-13.4)	0.345
**OS, probability (95% CI)**	82.2 (74.6–92.8)	78.3 (67.2-91.2)	95.7 (86.6-100)	0.104
**LFS, probability (95% CI)**	74.2 (63.6–84.3)	68.0 (56.2-82.2)	85.7 (72.0-100)	0.152
**GRFS, probability (95% CI)**	71.5 (53.3-79.3)	69.9 (52.1-77.9)	72.2 (54.0-86.5)	0.963
**EFS, probability (95% CI)**	78.8 (69.5-89.4)	72 (60.1-86.2)	95.2 (85.2-100)	0.046

Abbreviations: ATG, anti-thymocyte globulin; NHF, non–haplo-fever; HF, haplo-fever; CIs, cumulative incidence; aGvHD, acute graft-versus-host disease; cGvHD, chronic graft-versus-host disease; m/s, moderate to severe; CMV, cytomegalovirus; EBV, Epstein–Barr virus; MRD, measurable residual disease; NRM, non-relapse mortality; OS, overall survival; LFS, leukemia-free survival; GRFS, graft-versus-host disease–relapse–free survival; EFS, event-free survival.

Supplemental Table 7. Transplant outcomes of PTCy cohort.

**Transplant outcomes**	**PTCy cohort (n=72)**	**NHF group (n=31)**	**HF group (n=41)**	***P* value**
**CIs of aGvHD, incidence (95% CI)**				
I-IV°	27.9 (18.9-39.8)	16.1 (7.1-34.5)	36.8 (24.0-53.4)	0.02
II-IV°	19.4 (12.0-31.6)	6.5 (1.7-23.4)	29.3 (17.8-45.7)	0.01
III-IV°	6.9 (3.0-15.9)	6.5 (1.7-23.4)	7.3 (2.4-21.0)	0.86
**CIs of cGvHD, incidence (95% CI)**				
Total cGvHD	52.2 (38.3-63.0)	36.1 (15.0-51.9)	64.8 (45.6-77.3)	0.007
m/s cGvHD	26.9 (15.3-36.8)	21.8 (4.6-36.0)	31.0 (14.7-44.2)	0.322
**CIs of CMV reactivation, incidence (95% CI)**	62.5 (51.5-73.5)	61.3 (44.9-78.0)	63.4 (49.4-77.7)	0.89
**CIs of EBV reactivation, incidence (95% CI)**	37.7 (27.1-50.9)	38.9 (24.3-58.2)	35.7 (22.6-53.3)	0.74
**CIs of Relapse, incidence (95% CI)**	12.4 (2.8-21.1)	10.9 (0-21.8)	12.3 (0-23.1)	0.862
**CIs of MRD recurrence, incidence (95% CI)**	29.8 (18.2-39.8)	32.3 (13.6-46.9)	27.3 (12.1-39.9)	0.56
**NRM, incidence (95% CI)**	4.2 (0–8.8)	3.2 (0-9.2)	5.0 (0-11.5)	0.732
**OS, probability (95% CI)**	91.2 (84.8–98.2)	93.4 (85.0-100)	89.5 (80.3-99.8)	0.603
**LFS, probability (95% CI)**	68.4 (58.1-80.5)	67.7 (53.1-86.4)	69.8 (56.8-85.9)	0.775
**GRFS, probability (95% CI)**	62.6 (51.5-76.1)	61.1 (43.9-85.0)	63.2 (49.5-80.7)	0.732
**EFS, probability (95% CI)**	79.5 (70.3–89.9)	73.3 (56.9-94.5)	82.8 (71.9-95.3)	0.633

Abbreviations: PTCy, post-transplantation cyclophosphamide; NHF, non–haplo-fever; HF, haplo-fever; CIs, cumulative incidence; aGvHD, acute graft-versus-host disease; cGvHD, chronic graft-versus-host disease; m/s, moderate to severe; CMV, cytomegalovirus; EBV, Epstein–Barr virus; MRD, measurable residual disease; NRM, non-relapse mortality; OS, overall survival; LFS, leukemia-free survival; GRFS, graft-versus-host disease–relapse–free survival; EFS, event-free survival.

Supplemental Table 8. Baseline characteristics of patients in NHF and HF groups in the ATG cohort.

Characteristics	ATG cohort (n=71)	NHF group (n=50)	HF group (n=21)	*P* value
**Median age, years (range)**	48 (16-68)	48 (16-67)	44 (16-68)	0.71
**Gender, *n* (%)**				0.76
Male	42 (59.2)	29 (69.1)	13 (31.0)	
Female	29 (40.8)	21 (72.4)	8 (38.1)	
**Follow-up duration in days, median (range)**	929.5 (137-1322)	879.5 (137-1212)	1013 (179-1322)	<0.01
**Underlying disease, *n* (%)**				0.60
Myeloid disease	44 (72.1)	30 (60.0)	20 (40.0)	
Lymphoid disease	27 (38.0)	14 (66.7)	7 (33.3)	
**HCT-CI scores before allo-HSCT, *n* (%)**				0.69
0 (low risk)	52 (73.2)	36 (72.0)	16 (76.2)	
1–2 (intermediate risk)	14 (19.7)	11 (22.0)	3 (14.3)	
≥ 3 (high risk)	5 (7.0)	3 (6.0)	2 (9.5)	
**Conditioning, *n* (%)**				
MAC	134 (93.7)	48(96.0)	18 (85.7)	0.12
RIC	9 (6.3)	2(4.0)	3 (14.3)	
**Blood group disparity, *n* (%)**				0.62
Matched	26(56.5)	18(39.1)	8(17.4)	
Major mismatched	8(17.4)	5(10.9)	3(6.5)	
Minor mismatched	9(19.6)	5(10.9)	4(8.7)	
Major and minor mismatched	3(6.5)	1(2.2)	2(4.3)	
**DSA, *n* (%)**				0.25
Negative	38 (79.2)	23 (74.2)	15 (88.2)	
Positive	10 (20.8)	8 (25.8)	2 (11.7)	
**Disease status, *n* (%)**				0.60
Remission	62 (87.3)	43 (66.4)	19 (30.7)	
Active	9 (12.7)	7 (77.8)	2 (22.2)	

Abbreviations: ATG, anti-thymocyte globulin; NHF, non–haplo-fever; HF, haplo-fever; HCT-CI, Hematopoietic Cell Transplantation Comorbidity Index; allo-HSCT, allogeneic hematopoietic stem cell transplantation; MAC, myeloablative conditioning; RIC, reduced-intensity conditioning; DSA, donor-specific antibody.

Supplemental Table 9. Risk factors of HF in ATG cohort.

**Risk factors**	**Univariate Cox**		**Multivariate Cox**	
	**HR (95% CI)**	***P* value**	**HR (95% CI)**	***P* value**
Categorical variables				
**Gender match**				
Female_to_Male	Ref	Ref	Ref	Ref
Others	0.70 (0.11-4.41)	0.70	60.69 (0.01-4.4E6)	0.37
**Conditioning intensity**				
MAC	Ref	Ref	Ref	Ref
RIC	3.62 (0.20-64.59)	0.38	47.23 (0.00-8.1E6)	0. 43
**Diagnosis**				
Myeloid	1.00 (0.21-4.86)	1.0	0.00 (0.00-1.5E5)	0.41
Lymphoid	Ref	Ref	Ref	Ref
**Disease_status**				
Remission	1.43 (0.23-9.01)	0.70	6670.64 (0.00-4.6E10)	0.27
Active	Ref	Ref	Ref	Ref
Continuous variables				
**Age**	1.00 (0.95-1.05)	0.94	0.93 (0.76-1.14)	0.48
**Infused CD34 cells**	1.59 (0.87-2.91)	0.13	0.58 (0.02-18.59)	0.76
**Infused MNCs**	1.15 (0.97-1.36)	0.10	0.92 (0.52-1.63)	0.78
**Infused CD3**	5.73 (1.47-22.31)	0.01	0.00 (0.00-5.7E15)	0.44
**Infused CD4**	7.12 (1.06-47.92)	0.04	1.6E10(0.00-7.4E39)	0.50
**Infused CD8**	219.81(6.25-7732.98)	0.01	6.3E19 (0.00-1.7E57)	0.30
**Infused NK**	0.40 (0.00-56.51)	0.72	0.04 (0.00-5.4E6)	0.29
**Infused CD19**	25.43 (0.61-1061.88)	0.09	0.01 (0.00-3924)	0.46
**Infused $CD4^+$ TM**	75.17(1.44-3932.36)	0.03	2.0E13(0-3.4E34)	0.22

Abbreviations: HF, haplo-fever; ATG, anti-thymocyte globulin; MAC, myeloablative conditioning; RIC, reduced-intensity conditioning; MNC, mononuclear cells; $CD34^+$, cluster of differentiation 34 positive; CD3, cluster of differentiation 3 positive T cells; CD4, cluster of differentiation 4 positive T cells; CD8, cluster of differentiation 8 positive T cells; NK, natural killer cells; CD19, cluster of differentiation 19 positive B cells; $CD4^+$ TM, $CD4^+$ memory T cells.
